# Gut microbiota and risk of lower respiratory tract infections: a bidirectional two-sample Mendelian randomization study

**DOI:** 10.3389/fmicb.2023.1276046

**Published:** 2023-11-23

**Authors:** Wei Liu, Xinyan Wang, Ruizhi Feng, Chen Zhao, Jian Luo, Xiawei Zhang, Xuemei Liu, Mei Yang, Jie Min, Bing Mao, Hongli Jiang

**Affiliations:** ^1^Division of Pulmonary Medicine, Department of Internal Medicine, Institute of Integrated Traditional Chinese and Western Medicine, West China Hospital, Sichuan University, Chengdu, China; ^2^Department of Oral Medicine, Shanghai Engineering Research Center of Tooth Restoration and Regeneration, Stomatological Hospital and Dental School of Tongji University, Shanghai, China; ^3^Respiratory Medicine Unit and National Institute for Health Research, Nuffield Department of Medicine Experimental Medicine, Oxford Biomedical Research Centre, University of Oxford, Oxford, United Kingdom; ^4^Department of Pulmonary Diseases, State Key Laboratory of Biotherapy of China, West China Hospital, Sichuan University, Chengdu, China

**Keywords:** gut microbiota, lower respiratory tract infections, causal relationship, Mendelian randomization, *Blautia* genus

## Abstract

**Introduction:**

Observational studies have reported the association between gut microbiota and the risk of lower respiratory tract infections (LRTIs). However, whether the association reflects a causal relationship remains obscure.

**Methods:**

A bidirectional twosample Mendelian randomization (MR) analysis was conducted by assessing genome-wide association study (GWAS) summary statistics for gut microbiota taxa and five common LRTIs. MR methods including inverse-variance-weighted (IVW), MR-Egger, weighted median, simple mode, and weighted mode were used to analyze the causality. Gene pleiotropy was tested using MR-Egger regression and MR-PRESSO methods. Cochran’s Q test was used to check for heterogeneity. Leave-one-out analysis was used to assess the stability of effect sizes. Detected significant associations were validated by using an independent LRTI GWAS summary statistics dataset. An optional MR method of causal analysis using summary effect estimates (CAUSE) was further performed as a validation to avoid potential false-positive results.

**Results:**

According to the MR-Egger estimates in forward MR analysis, a causal effect of gut *Blautia* on increased odds of bronchiectasis and pneumonia was suggested. MR-Egger regression pleiotropy intercept methods detected no significant horizontal pleiotropy between the instrumental variables of these associations. MR-PRESSO global test examined no potential horizontal pleiotropy. Cochran’s Q test showed that no heterogeneity biased the results. The leave-one-out sensitivity analyses suggested robust causality results. These associations with consistent effect direction were successfully replicated in IVW analysis by using the validation GWAS dataset. However, these evidence of causality did not survive after applying strict Bonferroni correction or CAUSE analysis. The reverse MR analysis failed to achieve consistent results in the effect of LRTIs on gut microbiota through comprehensive discovery and validation processes.

**Discussion:**

This study established no strong causality between genetically predicted gut microbiome and the risk of lower respiratory tract infections. However, specific subtypes of microbial genera, such as *Blautia*, were identified as potential influencers and require further investigation, particularly at the species or strain levels.

## Introduction

Lower respiratory tract infections (LRTIs) remain a significant cause of morbidity and mortality worldwide, particularly affecting vulnerable populations such as the elderly, young children, and immunosuppressed individuals (Langelier et al., 2018). According to the Global Burden of Disease Study 2016, LRTIs were responsible for approximately 2,377,697 deaths globally in 2016, especially among people aged over 65 years (Collaborators, 2018). Despite substantial advances over the past decade, LRTIs still account for a fifth of all deaths worldwide, primarily in low- and middle-income countries. From an epidemiological point of view, definitions of LRTI mainly include bronchiectasis, pneumonia, influenza, acute bronchitis, and acute bronchiolitis (Collaborators, 2017). Treatment guidelines may vary among countries, but they generally focus on improving nutrition and hygiene, along with the distribution and usage of antimicrobial agents and vaccines (Kamata et al., 2022). Although early and appropriate antimicrobial treatment are available, many patients still succumb to LRTIs due to challenges such as drug resistance (Cazzola et al., 2017). Hence, there is a need for novel therapeutic strategies in addition to traditional antibiotics, making precision medicine for LRTIs an important approach.

In otherwise healthy individuals, the human gut is the most densely colonized organ, harboring an estimated 10^14^ bacteria from over 1,000 bacterial species (Bäckhed et al., 2005). Current studies have extensively explored the relationship between gastrointestinal microbiota and human diseases. Its importance in sustaining local and systemic tissue homeostasis has gained great recognition (Nenci et al., 2007; Gensollen et al., 2016). Furthermore, the metabolic by-products and ligands of gut commensal bacteria have the ability to modulate and fine-tune the development and function of the innate and adaptive immune system, which helps to protect against infections caused by diverse pathogens (Ichinohe et al., 2011; Abt et al., 2012). Studies taking advantage of germ-free mice and antibiotic-driven depletion of gut bacterial species have contributed significantly to the understanding of gut-lung axis in mediating a range of respiratory infectious diseases. For example, the disruption of gut microbiome development in infancy plays a role in increased susceptibility to pulmonary viral infections (Ichinohe et al., 2011) and the development of lung diseases including asthma and chronic obstructive pulmonary disease (Abrahamsson et al., 2014; Zeissig and Blumberg, 2014; Qu et al., 2022). Mechanically, gut microbes initiate the activation of the innate antiviral immune response via the interactions with pattern recognition receptors (ie., Toll like receptor) (Samuelson et al., 2015), and also contribute to the regulation of macrophage response and restoration of lung CD4^+^ and CD8^+^ T cells, which are crucial for improved survival in respiratory viral infections (Abt et al., 2012). Besides, imbalances in gut microbiome result in an exacerbation of cytokine-induced inflammation (Verdam et al., 2013), potentially resulting in lung morbidity. It is partially attributed to the reduced production of anti-inflammatory metabolites like short-chain fatty acids (SCFAs) (Trompette et al., 2014) and increased production of pro-inflammatory metabolites such as secondary bile acids (Duboc et al., 2013) from intestinal flora, which transported via circulation to play a role in gut-lung communication, thereby impacting respiratory health and infections. Probiotics and prebiotics have demonstrated effects in reducing the incidence of cystic fibrosis pulmonary exacerbations (Weiss et al., 2010), protecting against bacterial pneumonia (Vieira et al., 2016), and expediting recovery from respiratory viral infections (Kawahara et al., 2015; Samuelson et al., 2015). Understanding the role of gut microbiome and its influence on respiratory infections can bring further refinement to the discovery of biomarker and precision medicine for these diseases.

However, so far, it remains unclear whether there is a causal relationship between gut microbiome and LRTIs because of the confounding factors and possible reverse causation. Mendelian randomization (MR) is an emerging genetic epidemiological method that utilizes summary data from genome-wide association studies (GWASs) as instrumental variables to infer causality in exposure-outcome associations (Dan et al., 2021). It may help to identify specific causal microbe taxa to enhance the development of precision medicine in the treatment of LRTIs. Therefore, we employed a bidirectional two-sample MR analysis to investigate the causal effects of gut microbiome on the risk of five common LRTI diseases and vice versa.

## Materials and methods

**Figure 1:**
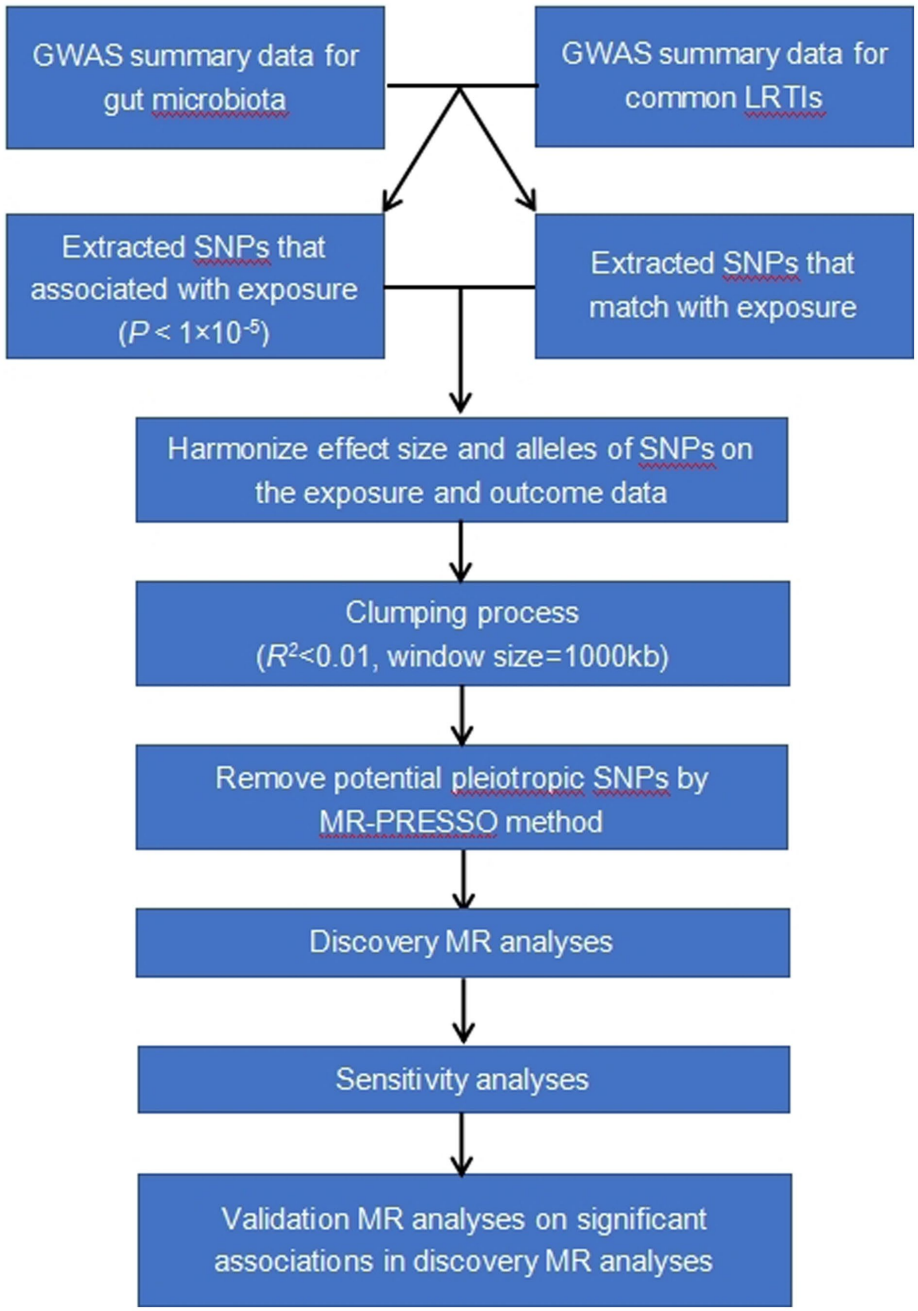
Study design and workflow.

The study methods were compliant with the STROBE-MR checklist (Skrivankova et al., 2021). Our analysis used publicly available GWAS summary statistics. No new data were collected, and no additional ethical approval or informed consent was required. The flowchart of the study was shown in Figure 1.

### Data sources

SNPs related to human 16S fecal microbiome composition were selected as instrumental variables from a large-scale multi-ethnic GWAS meta-analysis with 24 cohorts, comprising 18,340 individuals of different ethnicities and ages from the USA, Canada, Israel, South Korea, Germany, Denmark, the Netherlands, Belgium, Sweden, Finland, and the UK, most of whom had European ancestry (*n* = 13,266) (Kurilshikov et al., 2021). The microbial composition was profiled by targeting three distinct variable regions of the 16S rRNA gene, including V4 (10,413 samples, 13 cohorts), V3-V4 (4,211 samples, 6 cohorts), and V1-V2 (3,716 samples, 5 cohorts).

The discovery summary-level data of SNPs associated with the five common LRTI subtypes were obtained from the data of FinnGen Release 8 (released to the public on December 1, 2022),^1^ including bronchiectasis, acute bronchiolitis, acute bronchitis, influenza, and pneumonia. Cases were defined as participants with at least one inpatient or outpatient ICD-9/10 code as a primary diagnosis for these LRTIs. We limited LRTI data to samples of European ancestry to avoid potential bias aroused by population stratification. This GWAS consisted of 1,967 bronchiectasis cases (283,589 controls), 1,754 acute bronchiolitis cases (323,785 controls), 13,832 acute bronchitis cases (323,785 controls), 52,021 pneumonia cases (290,478 controls), and 7,580 influenza cases (286,619 controls).

The validation summary statistics of SNPs associated to the five common LRTI diseases were obtained from the GWAS reports measured in European participants from the UK Biobank by searching the GWAS catalog^2^ (Jiang et al., 2021), which included a total of 583 bronchiectasis cases (455,765 controls) (accession number: GCST90044075), 172 acute bronchiolitis and bronchitis cases (456,176 controls) (accession number: GCST90044069), 2,842 pneumonia cases (453,506 controls) (accession number: GCST90044067), and 248 influenza cases (456,100 controls) (accession number: GCST90044068).

We also examined the possibility of reverse causality of LRTIs on gut microbiome, using the discovery datasets of GWASs related to gut microbiome and LRTIs.

### Instrumental SNPs selection

The following selection criteria were used to choose the IVs: (1) SNPs associated with each genus were collected as potential IVs using a genome-wide significance threshold of *p* < 1 × 10^−5^ for their inclusion in the current study; (2) due to the presence of strong linkage disequilibrium (LD) among selected SNPs might bias the results, SNPs were clumped within study per bacterium genus using the PLINK (version 1.961) clumping procedure to eliminate the stringent LD between included IVs (*R^2^* < 0.01, with reference to the 1,000 Genomes Phase 3 CEU Project Panel (Abecasis et al., 2012), clumping window size = 1,000 kb) within a five megabase window; (3) to guarantee that the impact of SNPs on exposure corresponds to the same allele as the impact on the outcome, palindromic SNPs with intermediate allele frequencies were excluded; (4) when the exposure-related SNPs were not available in the outcome dataset, the proxy SNPs significantly correlated with the variants of interest were used (*R^2^* > 0.8); (5) selected SNPs with a minor allele frequency (MAF) ≤ 0.01 were excluded.

### Statistical analysis

A bidirectional two-sample MR analysis was conducted to evaluate the causal relationship between the gut microbiome and common LRTI subtypes. When only a single SNP was available to construct the IV, the ratio of coefficients method was used to obtain MR estimates with first-order weights used to generate standard errors. Where more than one SNP was available to construct the IVs for a given genus, the random effect inverse variance weighted (IVW) MR approach was used as the principal analysis to acquire an overall estimate of the causal effect. In addition, complementary methods including MR-Egger regression, weighted median, weighted model, MR-pleiotropy residual sum and outlier (MR-PRESSO), and simple model were used to examine the causal association. The MR-Egger regression is used to assess the presence of pleiotropy, which occurs when a genetic variant affects multiple traits or outcomes, violating the instrumental variable assumption of MR. It provides a robust estimate of the causal effect by accounting for pleiotropy and can detect and quantify any directional pleiotropy bias (Bowden et al., 2016b). The weighted median estimator is a robust approach to estimate the causal effect when there is heterogeneity in the causal estimates from different genetic variants. It selects the median estimate as the overall causal effect, giving more weight to instruments with smaller variances and providing valid results as long as at least 50% of the weight comes from valid instruments (Bowden et al., 2016a). Similar to the weighted median, the weighted mode estimator is also designed to handle heterogeneity. It selects the mode estimate as the causal effect, which is the most frequent estimate among valid instruments. This method is useful when there is more pronounced heterogeneity in the causal estimates (Hartwig et al., 2017). MR-PRESSO is a method used to identify and correct for outliers or genetic variants that violate the no pleiotropy assumption. It detects outliers and removes them from the analysis, allowing for a more accurate estimation of the causal effect. Additionally, MR-PRESSO provides an adjusted causal estimate after removing outliers (Verbanck et al., 2018). To account for multiple testing, a conservative Bonferroni threshold for statistical significance was set to *p* < 0.05 / 119 = 4 × 10^−4^ for the forward MR analysis and *p* < 0.05 / 5 = 1 × 10^−2^ for the reverse MR analysis.

We also employed a Bayesian posterior probabilities-based MR method namely Causal Analysis Using Summary Effect Estimates (CAUSE) (Morrison et al., 2020), as a further validation analysis for the associations replicated in both discovery dataset and validation dataset. This approach demonstrates a reduced susceptibility to false positive associations resulting from correlated and uncorrelated horizontal pleiotropy by utilizing the maximum independent SNPs to increase detection power. To include a larger number of IVs, LD pruning was performed using a threshold of *r*^2^ < 0.1 and *p* < 1 × 10^−3^ via a built-in function in the CAUSE R package, which utilized precomputed LD estimates.

### Sensitivity analysis

**Figure 2:**
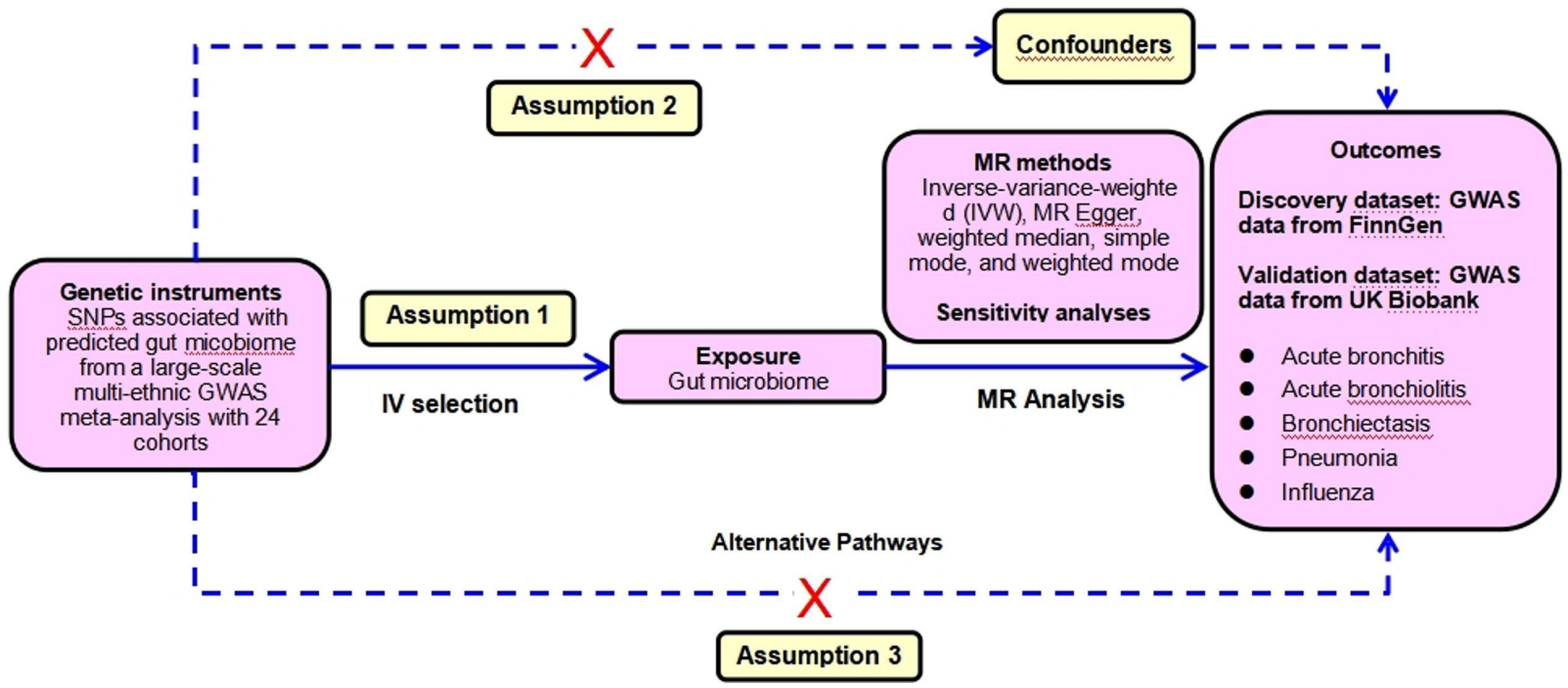
The SNP selection assumptions and forward MR analyses flowchart.

To ensure the reliability of the conclusion, sensitivity analyses were performed to verify whether heterogeneity and pleiotropy within the genetic variables could bias the MR results. First, the MR-Egger regression was applied to detect and adjust for the underlying horizontal pleiotropic effects among the selected IVs through the assessment of the intercept. Second, the MR-PRESSO global test of heterogeneity was conducted to identify the underlying horizontal pleiotropy. Third, the Cochran’ IVW Q statistics were used to quantify the heterogeneity across the selected IVs. With a consideration of possible significant heterogeneity between SNPs (*p* < 0.05), we applied a random-effect IVW model to perform the MR analysis. Fourth, the leave-one-out sensitivity analysis was implemented by omitting each instrumental SNP in turn to identify potential heterogeneous SNPs. Fifth, considering that various confounders including smoking, pulmonary comorbidities such as chronic obstructive pulmonary disease (COPD), asthma, lung fibrosis et al. were closely associated with the incidence of LRTIs, we conservatively queried each genus-related SNP used as the instrument in the PhenoScanner V2 database to identify SNPs that were significantly associated with GWAS traits potentially confounding LRTI phenotypes or might introduce horizontal pleiotropy at risk of affecting the five LRTI subtypes independent of gut microbiome at the genome-wide significance level. Whenever the GWAS *p*-value of the SNP was lower than the threshold (*p* < 1 × 10^−5^), we considered it to be correlated with the confounders (Kamat et al., 2019). We assessed the effect of gut microbiome after removing those SNPs from the MR estimates to exclude potential pleiotropic effects. This MR analysis was performed based on three assumptions: (1) The IV is closely associated with the exposure. (2) The IV is not associated with any potential confounders. (3) The IV can only influence the outcome via the exposure, and not by any other ways. The CAUSE method was used to detect the false positive error due to correlated horizontal pleiotropy. The SNPs selection assumptions and MR statistical analysis workflow were shown in Figure 2.

The strength of IVs was assessed by calculating the F-statistic using the formula *F* = *R*^2^ × (*N*-1-*K*) / (1-*R*^2^) × *K*, where *R*^2^ represented the proportion of variance in the exposure explained by the genetic variants, *N* represented sample size, and *K* represented the number of instruments (Palmer et al., 2012). *R*^2^ was calculated by using the formula *R*^2^ = 2 × MAF × (1–MAF) × beta^2^, where beta was the effect value of the genetic variant in the exposure, and MAF was the effect allele frequency of selected SNPs (Palmer et al., 2012; Burgess et al., 2016). If the corresponding F-statistic was >10, it was considered no significant weak instrumental bias was used. The power of the MR estimates was calculated using the mRnd MR power calculator^3^ (Burgess et al., 2017). MR analyses were performed using R (version 4.2.3), the TwoSampleMR package 0.5.6 (Hemani et al., 2018), MR-PRESSO (version 1.0) (Verbanck et al., 2018), and CAUSE (version 1.2.0) (Morrison et al., 2020).

## Results

### Forward MR analysis and sensitivity analyses

According to the selection criteria of IVs, a total of 1,517 SNPs associated with 119 bacterial genera were identified from the large-scale GWAS (Supplementary Table S1). After exclusion due to potential association to outcomes or outcome-related traits, unavailablility in outcome dataset (Supplementary Table S2), or palindrome, a total of 1,224 SNPs with four proxy SNPs were retained as IVs for the following forward MR analysis (Supplementary Table S3). The SNP number for bronchiectasis, acute bronchiolitis, acute bronchitis, influenza, and pneumonia in the discovery dataset was 1,220, 1,219, 1,221, 1,219, and 1,222, respectively. The main information of SNPs including effect allele, other allele, beta, SE, and *p* value were collected systematically for further analysis. The number of IVs associated with each bacterial genus varies from 3 to 20. The sum of F statistics for SNPs of individual bacterial genera was greater than the conventional threshold of 10, indicating that there was no significant bias from weak instrument variables (Table 1; Supplementary Table S4).

**Table 1:** MR results of causal effect of general gut microbiome on LRTI risk in forward analysis.

LRTIs	Datasets	Nsnp	Methods	F statistic	Beta	SE	*p*	OR (95% CI)	Horizontal pleiotropy			Heterogeneity	
									Intercept	SE	*p*	*Q*	*p*
Bronchiectasis	Discovery	1,220	MR-Egger	65.815	0.061	0.032	0.059	1.062 (0.998, 1.131)	−0.007	0.003	0.034	1215.693	0.624
			Weighted median		−0.005	0.019	0.786	0.995 (0.959, 1.032)					
			IVW		−0.002	0.012	0.864	0.998 (0.974, 1.022)					
			Weighted mode		−0.032	0.079	0.687	0.969 (0.829, 1.132)					
			Simple mode		−0.044	0.069	0.529	0.957 (0.835, 1.097)					
	Validation	1,076	MR-Egger	58.529	0.007	0.064	0.911	1.007 (0.888, 1.143)	0.004	0.006	0.519	1085.538	0.405
			Weighted median		0.081	0.038	0.031	1.084 (1.007, 1.167)					
			IVW		0.046	0.025	0.064	1.047 (0.997, 1.098)					
			Weighted mode		0.228	0.016	0.156	1.226 (0.917, 1.719)					
			Simple mode		0.228	0.136	0.093	1.226 (0.963, 1.638)					
Acute bronchiolitis	Discovery	1,219	MR-Egger	65.710	0.029	0.035	0.404	1.030 (0.961, 1.104)	−0.002	0.003	0.562	1268.436	0.236
			Weighted median		0.030	0.022	0.163	1.031 (0.988, 1.076)					
			IVW		0.011	0.014	0.436	1.011 (0.984, 1.038)					
			Weighted mode		0.091	0.082	0.271	1.095 (0.932, 1.287)					
			Simple mode		0.081	0.072	0.259	1.084 (0.942, 1.248)					
Acute bronchitis		1,221	MR-Egger	66.324	−0.020	0.013	0.105	0.980 (0.956, 1.004)	0.002	0.001	0.073	1288.878	0.131
			Weighted median		0.001	0.007	0.864	1.001 (0.987, 1.016)					
			IVW		0.000	0.005	0.937	1.000 (0.991, 1.010)					
			Weighted mode		0.022	0.029	0.466	1.022 (0.964, 1.083)					
			Simple mode		0.014	0.023	0.543	1.014 (0.969, 1.062)					
Acute bronchitis and bronchiolitis	Validation	1,233	MR-Egger	68.013	0.061	0.110	0.578	1.063 (0.857, 0.319)	−0.002	0.011	0.886	1256.368	0.308
			Weighted median		0.017	0.065	0.797	1.017 (0.895, 1.156)					
			IVW		0.047	0.042	0.270	1.048 (0.964, 1.138)					
			Weighted mode		−0.148	0.286	0.606	0.863 (0.492, 1.511)					
			Simple mode		−0.116	0.645	0.645	0.890 (0.544, 1.458)					
Influenza	Discovery	1,219	MR-Egger	66.144	0.003	0.017	0.848	1.003 (0.971, 1.037)	−0.001	0.002	0.446	1296.740	0.105
			Weighted median		−0.005	0.010	0.603	0.995 (0.977, 1.014)					
			IVW		−0.009	0.007	0.186	0.991 (0.979, 1.004)					
			Weighted mode		−0.027	0.040	0.499	0.973 (0.900, 1.052)					
			Simple mode		−0.012	0.033	0.719	0.988 (0.926, 1.054)					
	Validation	1,233	MR-Egger	68.013	−0.024	0.091	0.788	0.976 (0.817, 1.165)	0.006	0.009	0.518	1180.687	0.850
			Weighted median		0.027	0.052	0.602	1.027 (0.928, 1.137)					
			IVW		0.030	0.035	0.396	1.030 (0.962, 1.103)					
			Weighted mode		0.031	0.230	0.894	1.031 (0.657, 1.617)					
			Simple mode		0.056	0.194	0.775	1.057 (0.723, 1.546)					
Pneumonia	Discovery	1,222	MR-Egger	66.324	0.001	0.007	0.897	1.001 (0.987, 1.015)	−0.000	0.001	0.754	1364.909	0.005
			Weighted median		−0.003	0.004	0.484	0.997 (0.989, 1.005)					
			IVW		−0.001	0.003	0.681	0.999 (0.993, 1.004)					
			Weighted mode		−0.005	0.015	0.733	0.995 (0.966, 1.025)					
			Simple mode		−0.005	0.015	0.733	0.995 (0.966, 1.025)					
	Validation	1,233	MR-Egger	68.013	−0.023	0.027	0.400	0.977 (0.927, 1.031)	0.003	0.003	0.236	1176.692	0.735
			Weighted median		0.007	0.015	0.664	1.007 (0.977, 1.037)					
			IVW		0.007	0.010	0.510	1.007 (0.987, 1.028)					
			Weighted mode		0.013	0.063	0.840	1.013 (0.895, 1.147)					
			Simple mode		−0.001	0.056	0.982	0.999 (0.895, 1.115)					

Nsnp is the number of SNPs used to estimate the causal effect size.

According to the five MR methods, the genetically determined gut microbiome, as a whole, had no causal relationship with LRTIs. The results of Cochran’s IVW Q test showed no significant heterogeneity of these IVs. The discovery findings were confirmed in independent validation MR analysis (Table 1). Detailed information and strengths of IVs in validation dataset were presented in Supplementary Tables S5, S6.

However, the results of MR analyses for individual gut microbial genus in discovery datasets revealed that genetically predicted relative abundance of specific genera might be causally associated with an increased or decreased risk of LRTI (Supplementary Table S7). For example, based on the IVW estimates, *Bifidobacterium* was negatively associated to the risk of bronchiectasis [odds ratio (OR): 0.688, 95% confidence interval (CI): 0.520–0.910, *p* = 0.009], but positively associated to the risk of influenza (OR: 1.225, 95% CI: 1.061–1.415, *p* = 0.006). While according to the MR-Egger analysis, *Blautia* was consistently correlated with an increased risk of bronchiectasis (OR: 2.956, 95% CI: 1.143–7.648, *p* = 0.049), influenza (OR: 1.856, 95% CI: 1.140–3.022, *p* = 0.032), and pneumonia (OR: 1.336, 95% CI: 1.036–1.724, *p* = 0.049). In addition, abundance of genera *Erysipelatoclostridium* acted as a risk factor for acute bronchiolitis (OR: 3.483, 95% CI: 1.266–9.583, *p* = 0.031) and influenza (OR: 1.764, 95% CI: 1.089–2.860, *p* = 0.038), while *Oxalobacter* acted as a protective factor for acute bronchiolitis (OR: 0.799, 95% CI: 0.653–0.978, *p* = 0.030) and bronchitis (OR: 0.913, 95% CI: 0.842–0.989, *p* = 0.027) (Supplementary Table S8; Supplementary Figures S1, S2).

For these identified associations, the Cochran’s Q test suggested that there was no significant heterogeneity across all these selected genetic instruments (Supplementary Table S8). The horizontal pleiotropy was evaluated by MR-Egger regression, and the results indicated that there was evidence of potential horizontal pleiotropy to skew the influence of *Anaerostipes* on bronchiectasis (*p* = 0.002), *Erysipelatoclostridium* on acute bronchiolitis (*p* = 0.030) and influenza (*p* = 0.041), *Holdemania* (*p* = 0.010) and *Olsenella*on (*p* = 0.035) on acute bronchiolitis, *Oscillospira* on acute bronchitis (*p* = 0.038), and *Ruminococcus_2* on pneumonia (*p* = 0.039) (Supplementary Table S8). The MR-Egger intercept test was sensitive to outliers and violations of INstrument Strength Independent of Direct Effect assumption, thus less efficient. Therefore, we also conducted MR-PRESSO global test, which was more robust to outliers. Nevertheless, no sign of horizontal pleiotropy was detected and no outliers were found by MR-PRESSO test (Supplementary Table S9). The leave-one-out analysis showed that no single SNP was driving these significant MR estimates, indicating that the results of the current MR analysis were robust (Supplementary Figure S3). However, after Bonferroni correction for multiple tests, none of the significant associations presented between genetically predicted gut microbiome with LRTIs.

**Figure 3:**
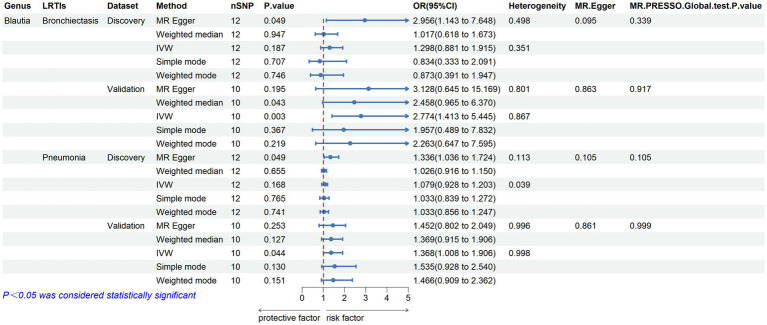
Validated casual links between specific gut microbial genus and LRTIs in forward MR analyses.

Furthermore, to validate the causal relationship identified in the discovery sample set, summary statistics from independent LRTI GWASs were employed. The validation MR analysis solely confirmed the causal association with the same direction between genetically predicted enrichment of microbial genus *Blautia* with bronchiectasis (IVW, OR: 2.774, 95% CI: 1.413–5.445, *p* = 0.003) and pneumonia (IVW, OR: 1.386, 95% CI: 1.008–1.906, *p* = 0.044) (Table 2; Figure 3). Still, application of the Bonferroni correction did not yield any statistically significant differences in validation. To further confirm the validated casual links between *Blautia* and LRTIs, CAUSE analysis was then performed. However, it indicated that the sharing model was better than the causal model, without providing consistent significant causality (Supplementary Table S10).

**Table 2:** MR results of significant causal effect of gut microbiome on LRTI risk in forwad analysis (*p* < 0.05).

Genus	LRTI	Dataset	N snp	Methods	Beta	SE	*p*	OR (95% CI)	Horizontal pleiotropy			Heterogeneity	
									Intercept	SE	*p*	*Q*	*p*
*Blautia*	Bronchiectasis	Discovery	12	MR-Egger	1.084	0.485	**0.049**	2.956 (1.143, 7.648)	−0.062	0.034	0.095	8.785	0.498
				Weighted median	0.017	0.254	0.947	1.017 (0.618, 1.673)					
				IVW	0.261	0.198	0.187	1.298 (0.881, 1.915)				12.175	0.351
				Simple mode	−0.181	0.469	0.707	0.834 (0.333, 2.091)					
				Weighted mode	−0.136	0.409	0.746	0.873 (0.391, 1.947)					
		Validation	10	MR-Egger	1.141	0.805	0.195	3.128 (0.645, 15.169)	−0.011	0.061	0.863	4.585	0.801
				Weighted median	0.908	0.482	0.043	2.458 (0.965, 6.370)					
				IVW	1.020	0.344	**0.003**	2.774 (1.413, 5.445)				4.613	0.867
				Simple mode	0.672	0.707	0.367	1.957 (0.489, 7.832)					
				Weighted mode	0.817	0.618	0.219	2.263 (0.674, 7.595)					
	Pneumonia	Discovery	12	MR-Egger	0.290	0.130	**0.049**	1.336 (1.036, 1.724)	−0.016	0.009	0.105	15.558	0.113
				Weighted median	0.026	0.058	0.655	1.026 (0.916, 1.150)					
				IVW	0.076	0.055	0.168	1.079 (0.928, 1.203)				20.517	0.039
				Simple mode	0.033	0.106	0.765	1.033 (0.839, 1.272)					
				Weighted mode	0.033	0.096	0.741	1.033 (0.856, 1.247)					
		Validation	10	MR-Egger	0.372	0.303	0.253	1.452 (0.802, 2.627)	−0.004	0.022	0.860	1.265	0.996
				Weighted median	0.314	0.206	0.127	1.369 (0.915, 2.049)					
				IVW	0.327	0.162	**0.044**	1.386 (1.008, 1.906)				1.298	0.998
				Simple mode	0.429	0.257	0.130	1.535 (0.928, 2.540)					
				Weighted mode	0.382	0.244	0.151	1.466 (0.909, 2.362)					

Nsnp is the number of SNPs used to estimate the causal effect size; Bold values denote statistical significance at the *p* < 0.05 level.

### Reverse MR analysis and sensitivity analyses

We further examined the causal effect of LRTI diseases on gut microbiome by reverse MR analyses. The detailed information and predictive power of selected IVs were presented in Supplementary Tables S11–S13. The conventional MR methods resulted in multiple causal relationships between LRTI and gut microbiome (Supplementary Table S14). Specifically, according to the results of bidirectional MR analyses based on the discovery dataset, we found a bidirectional causal association between *Escherichia_Shigella* (Forward IVW, OR: 0.851, 95% CI: 0.737–0.982, *p* = 0.028; Reverse IVW, OR: 0.851, 95% CI: 0.763–0.950, *p* = 0.004) and acute bronchitis, as well as *Eubacterium_fissicatena_group* (Forward IVW, OR: 0.897, 95% CI: 0.816–0.987, *p* = 0.025; Reverse IVW, OR: 0.793, 95% CI: 0.647–0.971, *p* = 0.025) and acute bronchitis (Supplementary Tables S8, S15).

The Bonferroni correction was also performed in reverse MR analysis to account for five exposures, where *p* < 0.01 (0.05/5) was defined as a statistically significant difference. The causal effect of Bronchiectasis on *Corprobacter* (Reverse MR IVW, *p* = 0.008) and *Ruminiclostridium_6* (Reverse MR IVW, *p* = 0.007), acute bronchiolitis on *Corprobacter* (Reverse MR IVW, *p* = 0.006) and *Eggerthella* (Reverse MR IVW, *p* = 0.003), acute bronchitis on *Escherichia_Shigella* (Reverse MR IVW, *p* = 0.004), *Oscillibacter* (Reverse MR IVW, *p* = 0.002), and *Veillonella* (Reverse MR IVW, *p* = 0.001), as well as pneumonia on *Haemophilus* (Reverse MR IVW, *p* = 0.003) was still significant after Bonferroni correction. There was no evidence of heterogeneity between IV estimates with IVW methods from individual SNPs and no pleiotropy effect for these detected associations. No pleiotropic outliers were detected according to the MR-PRESSO tests (Supplementary Table S16). However, as there were not enough IVs used in the MR to undertake a sensitivity analysis for the causal effect of acute bronchiolitis on most microbial genera, the results should be interpreted with caution. To verify these causalities, we also conducted validation MR analysis by using independent LRTI GWAS data. Nevertheless, none of the associations were replicated in the validation dataset, indicating a poor causal effect of LRTIs on the gut microbiome.

## Discussion

Colonization of the intestine with commensal bacteria is known to play a major role in the maintenance of the integrity of lung tissues against foreign infections. Intestinal flora has a powerful direct and indirect regulatory effect on the human immune system by increasing the number of immune cells, producing SCFAs and immunoglobulins, enhancing oral tolerance, and controlling inflammation (Samuelson et al., 2015). Consequently, an altered gut microbiome is always associated with various ensuing diseases including respiratory diseases. In this study, using the summary statistics of gut microbiota from the largest GWAS report and the summary statistics of LRTIs from the FinnGen consortium R8 release data and UK Biobank, we performed a bidirectional two-sample MR analysis to refer the causal association between gut microbiota and five common phenotypes of LRTI, which might be helpful to shed light on the impact of gut microbiota on airway immunity and the host’s ability to defend against respiratory infections. Additionally, our findings may inspire the development of precision medicine for treating these scenarios.

Through a comprehensive discovery and validation approach, this study suggested *Blautia* as a potential risk factor for an increased risk of bronchiectasis and pneumonia. Despite the causality did not surpass the strict Bonferroni correction threshold and was not confirmed in CAUSE analysis, it provided suggestive evidence of a potential causal effect between genus *Blautia* and LRTI frequency and might stimulate further specialized studies to gain insights into its impact on respiratory health. As a dominant genus of anaerobic bacteria in the feces and intestines of mammals, the probiotic characteristics of *Blautia* and its protective role in various host physiological dysfunctions have been reported, such as obesity, diabetes, cancer, and inflammatory bowel diseases. *Blautia* contributes to maintaining environmental balance in the intestine, preventing inflammation by upregulating intestinal regulatory T cells, and producing SCFAs (Kim et al., 2014). Its ability to produce bacteriocin, one of the common secondary metabolites that possess antibacterial activity against pathogenic microorganisms such as *Listeria monocytogenes*, *Clostridium perfringens*, and *Escherichia coli* (Martinez et al., 2013), also gives *Blautia* the potential to inhibit the colonization of pathogenic bacteria in the intestine (Liu et al., 2021). Our results did not align with previous findings regarding the protective effect of *Blautia* in human diseases. However, higher abundance of *Blautia* was also reported in the fecal microbiota of patients with irritable bowel syndrome, ulcerative colitis, functional gastrointestinal symptoms (Rajilić-Stojanović et al., 2011; Nishino et al., 2018; Ohlsson, 2022), and breast cancer (Luu et al., 2017), suggested its association to the local or systemic inflammation. Indeed, *Blautia*, especially *Blautia coccoides*, activates the secretion of inflammatory cytokines such as tumor necrosis factor α (TNF-α) to an even greater extent than lipopolysaccharide (Tuovinen et al., 2013). TNF-α is a potent protective cytokine that contributing to anti-viral and anti-bacterial responses during the early phase of infection, however, excessive production causes heightened lung immunopathology and inflammation, particularly in the late phase of infection. Besides, in patients with rheumatoid arthritis, increased relative abundance of gut genus *Blautia* was related with lower levels of T cells, B cells, CD4^+^ T cells, and Tregs (Li et al., 2021). The findings suggest that *Blautia* species act as culprits in the pathogenesis of infectious diseases potentially due to their pro-inflammatory and anti-immune properties. Mediterranean diet (MD) is plant-based and consistently considered to be benefit on human health (Barber et al., 2023). Studies demonstrated that administration of MD reduced *Blautia* within gut microbiota (Merra et al., 2020; Zhu et al., 2020). Evidence for the effects of dietary fiber within MD showed a direct suppressive effect on *Clostridium difficile* infection. As previous study has indicated a positive association of *Blautia* with key inflammatory cytokines such as TNF-α, the authors hypothesized that MD improves the inflammatory milieu and infection through modulation of gut microbiota, at least in part (Merra et al., 2020; Zhu et al., 2020). The conflicting conclusions may be related to the genomic difference within the genus, as there are 12 independent *Blautia* species with a total of 195 genome assemblies, nevertheless, most studies focused on the genus level and did not delve into investigations at species or even strain-levels (Liu et al., 2021). Therefore, drawing general conclusions at the genus level may lead to partial understandings and misinterpretations because different species of *Blautia* may exert different effects on human health. For example, *Blautia coccoides* was reported to be positively associated to the level of cytokines including TNF-α (Tuovinen et al., 2013), while *Blautia luti* and *B. wexlerae* in the gut microbiota of obese children was negatively related to those proinflammatory cytokines and chemokines (Benítez-Páez et al., 2020).

Although not consistently validated, our results implied that specific genera of gut commensal microbiota compositions might have protective effects against respiratory infections, highlighting their potential use as medications in the context of airway infections (Wolvers et al., 2010; Ozen et al., 2015). Based on the results from the discovery dataset, an increased abundance of *Oxalobacter* was consistently related to a lower risk of acute bronchiolitis and bronchitis, which are primarily caused by viral infections, particularly respiratory syncytial virus (RSV) (Kinkade and Long, 2016; Joseph and Edwards, 2019). *Oxalobacter* is a Gram-negative bacterium that degrades oxalate in the gut to decrease urinary oxalate excretion. Its probiotics preparation has been commercially available as a biotherapeutic agent in the management of calcium oxalate renal stones (Hiremath and Viswanathan, 2022). *Oxalobacter* was identified to be associated with intestinal virus infection (Gozalbo-Rovira et al., 2021) and infectious urinary stone (Bruyere et al., 2008). Our analyses gave a new clue that use of *Oxalobacter* strains might show beneficial effects on the host immunity and/or against pathogens in respiratory system. *Bifidobacterium* is a genus of Gram-positive, anaerobic bacteria that are commonly found in the human gut microbiome. These bacteria are known for their immunomodulatory properties and have been shown to have a beneficial impact on human health (Hidalgo-Cantabrana et al., 2017). In our study, *Bifidobacterium* was suggested as a protective factor for the risk of bronchiectasis, which is associated to bacteria-related recurrent respiratory tract infections (RRTIs) (Amati et al., 2019). Studies have revealed that RRTI patients suffer from intestinal flora imbalance (Ozen et al., 2015; Li et al., 2019), manifested as a significant reduction in the number of *Bifidobacteria* (Peng et al., 2016; Li et al., 2019). Restoring *Bifidobacteria* with oral *Bifidobaeterium tetravaccine* tablets (Live) effectively maintained the balance of intestinal micro-ecology and reduced average annual frequency of acute respiratory tract bacterial infection and use of antibiotics (Li et al., 2019). Thus, *Bifidobacterium* may be viewed as potential next-generation probiotic candidates in the treatment of bacterial lung infections. Actually, studies evaluating the use of *Bifidobacterium* as probiotics have already demonstrated their effect in the control of respiratory viral infections, such as COVID-19 (Taufer and Rampelotto, 2023) and H7N9 (Zhang et al., 2020). The potential mechanisms of action underlying the protective effects of *Bifidobacterium*, drawing from current knowledge, may involve the stimulation on immune system, reduction of inflammation, competitive advantage with pathogenic microbes, and maintenance of gut barrier function (Taufer and Rampelotto, 2023). A group of SCFAs-producing bacteria including *Parabacteroides* (Ahmed et al., 2019), *Anaerotruncus* (Yan et al., 2019), and *Lachnospiraceae_NC2004_group* (Egerton et al., 2022) were also implied to be protective in LRTIs by our results. SCFAs, mostly acetic acid, propionic acid, and butyric acid, are metabolites produced after gut microbial fermentation of dietary fiber. Accumulated evidence supported that SCFAs not only maintain local and systemic immune homeostasis but also boost host immunity to pathogens in a range of airway inflammatory conditions (Antunes et al., 2023; Dang et al., 2023), through sophisticated modulations on the maturation, accumulation, and function of immune cells, activating the transmembrane G protein-coupled receptors, and inhibiting histone deacetylases, et al. (Tan et al., 2014).

Notably, the association between gut microbiome and LRTIs found in the observational studies may be influenced by reverse causation. To address it, we performed a reverse MR analysis to investigate the causation in the opposite direction. Although there were some initial indications of causal effects of LRTIs on the gut microbiome in the discovery sample set, no notable genetically predicted associations were observed in the validation dataset. This suggested that reverse causation is unlikely to explain the findings in forward MR analysis.

This study has several strengths. To the best of our knowledge, this is the first MR study to infer the causal relationship between the gut microbiota and LRTIs. Genetic variants used to represent the gut microbiota were sourced from the largest available GWAS analysis, ensuring the strength of instruments for MR approach. Horizontal pleiotropy was detected and excluded by using the MR-PRESSO and MR-Egger regression intercept term tests. Furthermore, we conducted a comprehensive validation process by using an independent sample data and the CAUSE method to facilitate robust causal inferences. Our study somehow deepens the understanding of the gut microbiome on human health and highlights the microbiome-related agents as potential precision therapeutics to ensure enhanced resistance toward respiratory infections.

There are several limitations that should be considered while interpreting the results of this study. First, a standard MR method assumed a linear relationship between exposure and outcome, so the non-linear association and threshold effect between gut microbiota and LRTIs could not be detected. Second, we only explored the causal links between gut microbiota and LRTIs at the taxonomic level of genus, thus their associations at the species level could not be revealed. Third, although most participants in the GWAS meta-analysis for gut microbiota data were of European descent and the outcome GWAS data were restricted only from European subjects, the interference from population stratification might still exist. Moreover, the extrapolation of the current findings to other ethnic groups was limited. Future MR studies on this topic should be considered in non-European populations to confirm the results. Fourth, although we measured LD among all selected SNPs using northern Europeans from Utah samples from the 1,000 Genomes Project, we could not exclude the possibility that our results might be affected by unmeasured confounders. Fifth, by selecting representative diseases for LRTIs, we aimed to capture a range of lower respiratory tract infections of clinical importance. The ambiguous and broad phenotype definition for LRTIs might induce the risk of non-differential mismatch between specific genera and individual diseases because high heterogeneities in LRTIs due to varied etiologies, pathogens, disease stages, clinical and radiology features, and physiology/lung functions might significantly influence the results. For example, the inclusion of influenza with a blurred boundary between upper and lower respiratory tract effects potentially introduced a potential limitation in the causal inferences drawn from our analysis. Sixth, since the data sources did not include individual level data, we failed to report the number of individuals at each stage of the study and the reasons why individuals were excluded from further study. This limitation might influence the adjustment of validity and generalisability of results by readers. Finally, the genetic instruments used in the MR analyses still might have pleiotropic effects, which could produce spurious findings. To limit this risk, we conducted multiple sensitivity analyses to provide statistical evidence of bias from pleiotropy or genetic confounding. But still, since the results failed to meet the stringent threshold in Bonferroni correction and CAUSE method, it is important to interpret our findings with caution, particularly when making specific causal inferences related to individual diseases within the LRTI group.

## Conclusion

In summary, this study does not provide robust evidence for a causal relationship between genetically predicted gut microbiome genera and common phenotypes of lower respiratory tract infections. However, specific genera of gut commensal microbiota, such as *Blautia*, *Oxalobacter*, *Bifidobacterium*, and several SCFA-producing bacteria, are suggested to be potential indicators for respiratory infection susceptibility. Further specialized investigations, particularly studies focusing on the species or strain levels, are needed to gain more insights into their impact on lung health and to unlock the potential use of gut microbiota-based immune-regulatory therapies in respiratory infectious diseases.

## Data availability statement

The datasets presented in this study can be found in online repositories. The names of the repository/repositories and accession number(s) can be found in the article/supplementary material.

## Author contributions

WL: Conceptualization, Funding acquisition, Investigation, Supervision, Writing – original draft, Writing – review & editing. XW: Formal analysis, Methodology, Writing – original draft. RF: Formal analysis, Methodology, Writing – original draft. CZ: Formal analysis, Methodology, Writing – original draft. JL: Data curation, Formal analysis, Methodology, Writing – original draft. XZ: Data curation, Formal analysis, Writing – original draft. XL: Data curation, Methodology, Software, Writing – original draft. MY: Formal analysis, Software, Writing – original draft. JM: Data curation, Methodology, Writing – original draft. BM: Formal analysis, Supervision, Writing – review & editing. HJ: Funding acquisition, Supervision, Writing – review & editing.

## Footnotes

^1^ https://finngen.gitbook.io/documentation/

^2^ https://www.ebi.ac.uk/gwas/

^3^ https://cnsgenomics.com/shiny/mRnd/

## Glossary

AMPs: Antimicrobial peptides
CAUSE: Causal Analysis Using Summary Effect Estimates
COPD: Chronic obstructive pulmonary disease
CI: Confidence interval
GWAS: Genome-wide association study
IV: Instrumental variable
IVW: Inverse variance weighted
LD: Linkage disequilibrium
LRTIs: Lower respiratory tract infections
MR: Mendelian randomization
MD: Mediterranean diet
OR: Odds ratio
RRTIs: Recurrent respiratory tract infections
RSV: Respiratory syncytial virus
SCFAs: Short-chain fatty acids
SNP: Single nucleotide polymorphism
TNF-α: Tumor necrosis factor α
