# Supplementary material for: Gut microbiota and risk of lower respiratory tract infections: a bidirectional two-sample Mendelian randomization study
Source: Front Microbiol. 2023 Nov 23;14:1276046. doi: 10.3389/fmicb.2023.1276046 (PMC10702245; doi:10.3389/fmicb.2023.1276046)
Supplement: Supplementary file 1 [file Data_Sheet_1.zip › Supplementary Figures.docx]

| **Bronchiectasis** | | |
| --- | --- | --- |
| *Bifidobacterium* | *Eubacterium_brachy_group* | *Eubacterium_ventriosum_group* |
| 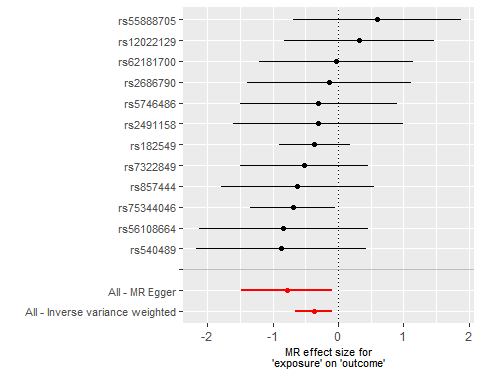 | 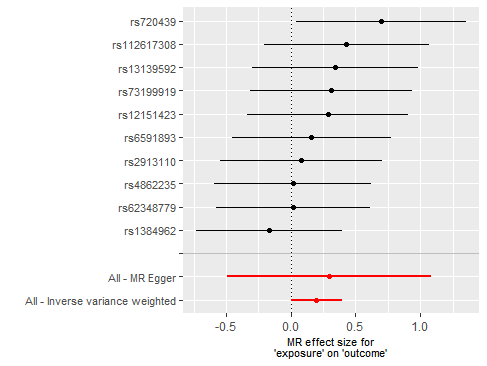 | 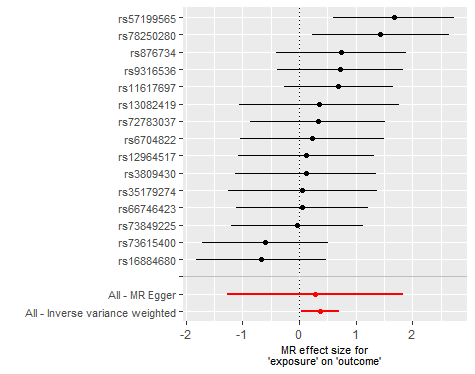 |
| *LachnospiraceaeFCS020_group* | *Parabacteroides* | *Peptococcus* |
| 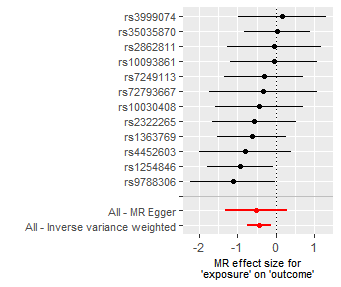 | 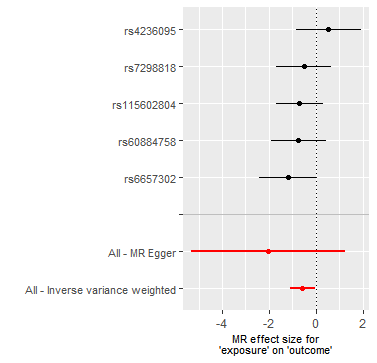 | 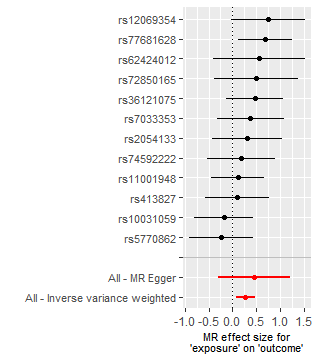 |
| **Acute bronchiolitis** | | |
| *Clostridium_sensu_stricto* | *Faecalibacterium* | *Haemophilus* |
| **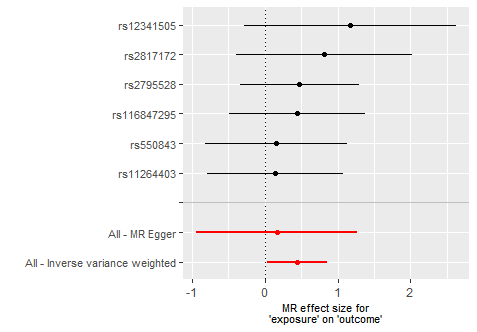** | **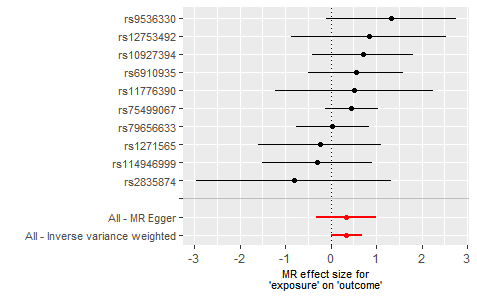** | **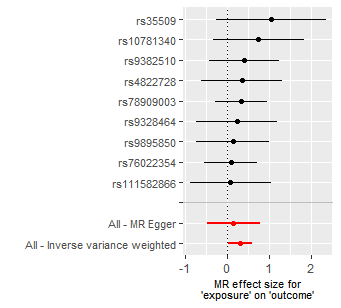** |
| *Oxalobacter* | *Tyzzerella* | *Veillonella* |
| 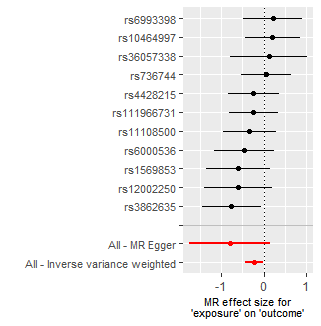 | 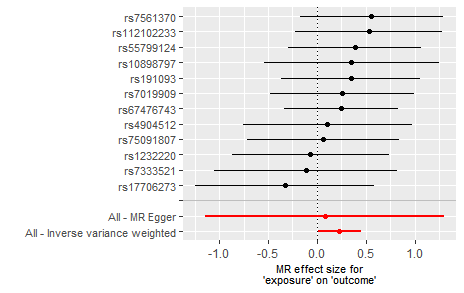 | 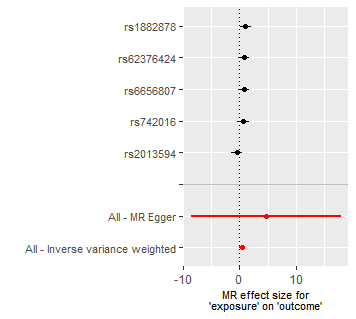 |
| **Acute bronchitis** | | |
| *Escherichia_Shigella* | *Eubacterium_fissicatena_group* | *Oxalobacter* |
| **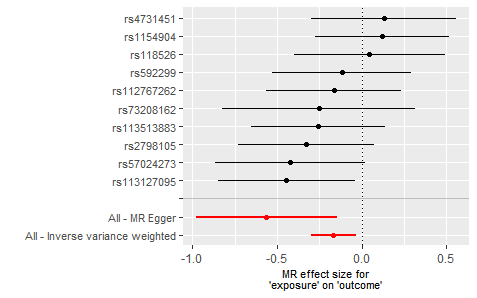** | **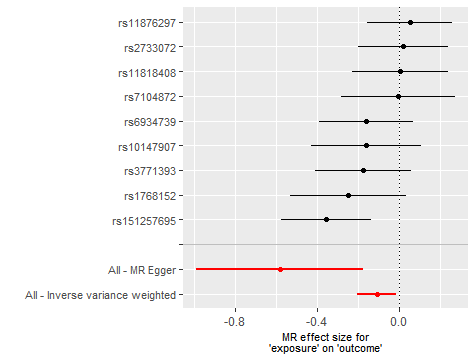** | **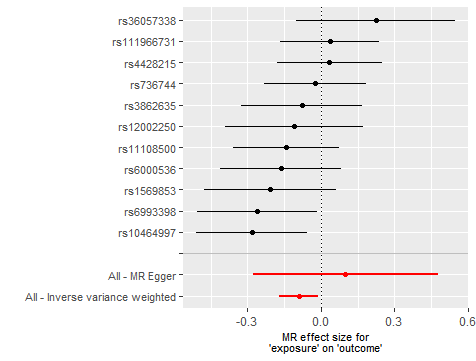** |
| **Influenza** | | |
| *Bifidobacterium* | *Lachnoclostridium* | *Prevotella_9* |
| **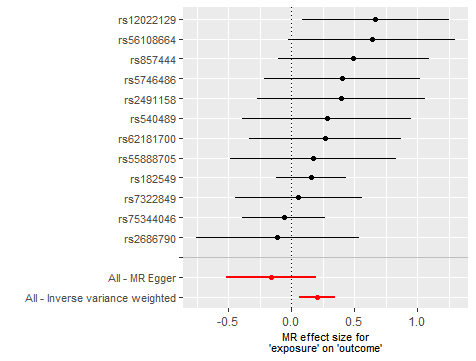** | **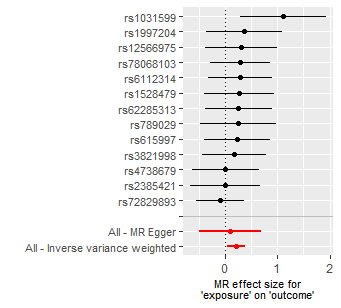** | **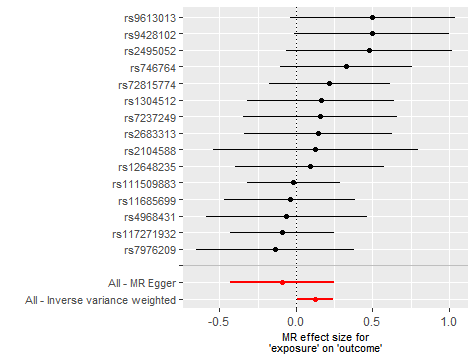** |
| **Pneumonia** | | |
| *Anaerotruncus* | *Barnesiella* | *Lachnospiraceae_NC2004_group* |
| **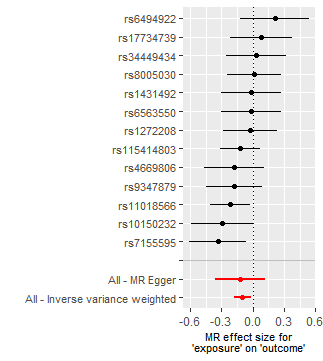** | **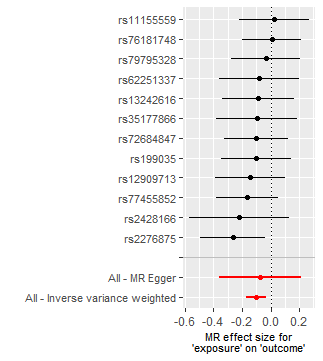** | **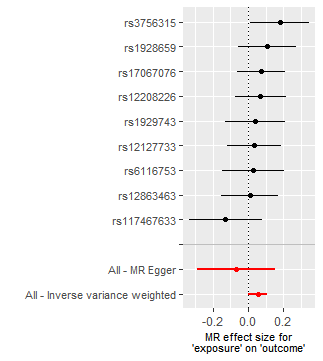** |

Supplementary Figure S1. Forest plots for the causal association between gut microbiota and LRTIs in forward MR analysis of discovery dataset.

| **Bronchiectasis** | | |
| --- | --- | --- |
| *Bifidobacterium* | *Eubacterium_brachy_group* | *Eubacterium_ventriosum_group* |
| 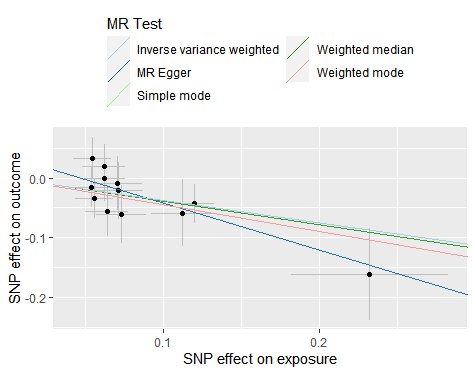 | 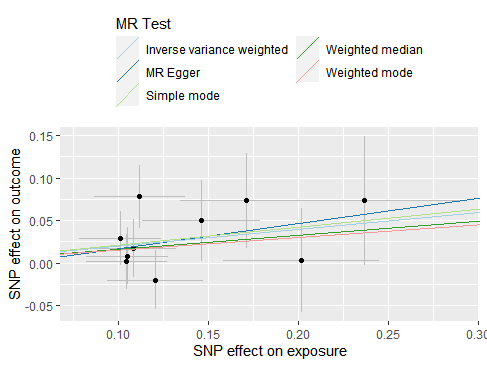 | 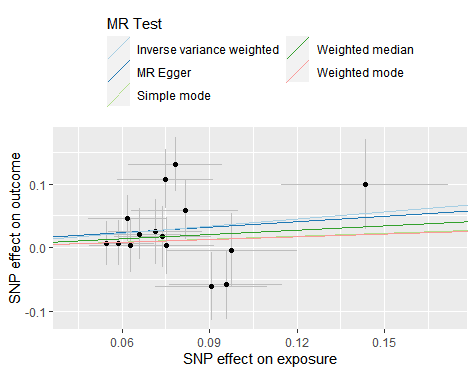 |
| *LachnospiraceaeFCS020_group* | *Parabacteroides* | *Peptococcus* |
| 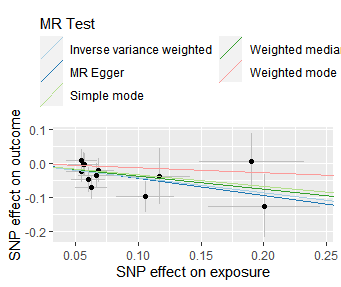 | 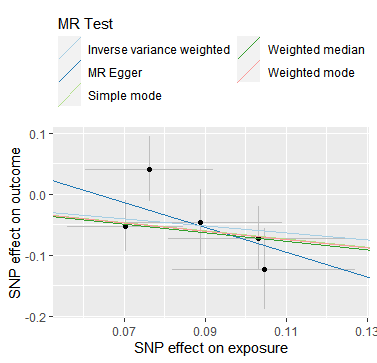 | 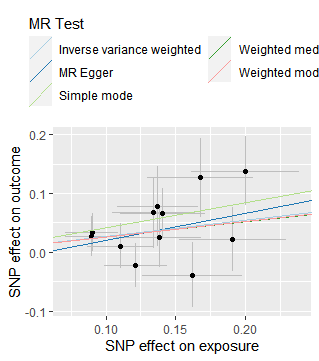 |
| **Acute bronchiolitis** | | |
| *Clostridium_sensu_stricto* | *Faecalibacterium* | *Haemophilus* |
| **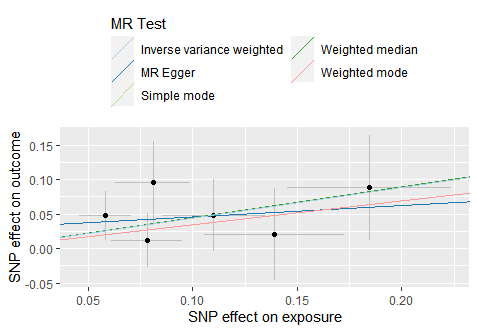** | **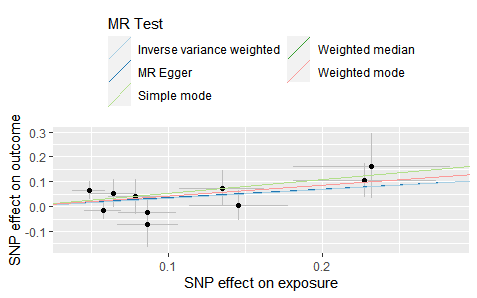** | **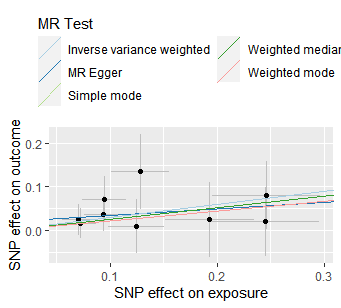** |
| *Oxalobacter* | *Tyzzerella* | *Veillonella* |
| 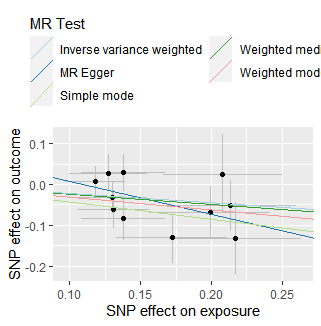 | 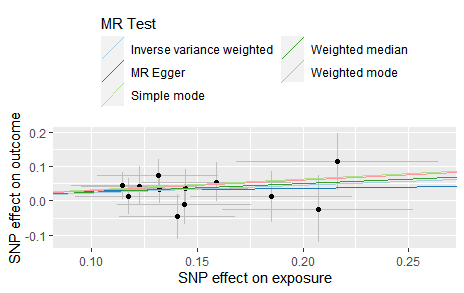 | 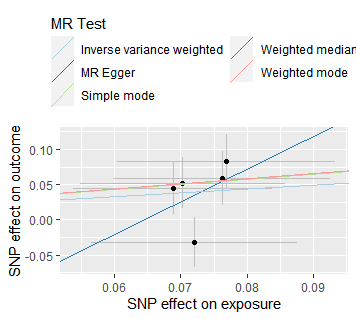 |
| **Acute bronchitis** | | |
| *Escherichia_Shigella* | *Eubacterium_fissicatena_group* | *Oxalobacter* |
| **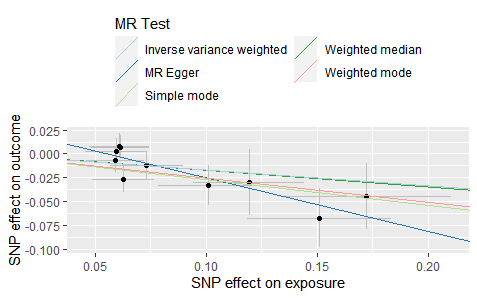** | **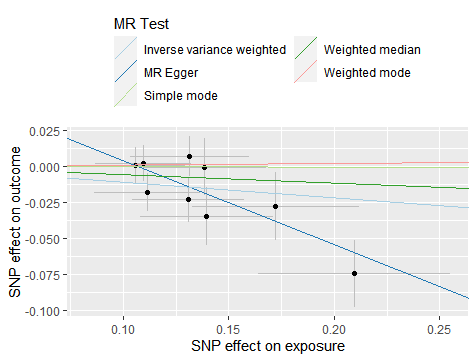** | **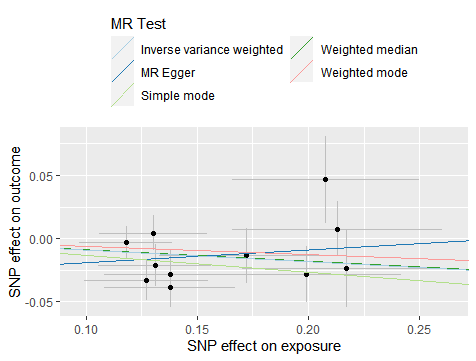** |
| **Influenza** | | |
| *Bifidobacterium* | *Lachnoclostridium* | *Prevotella_9* |
| **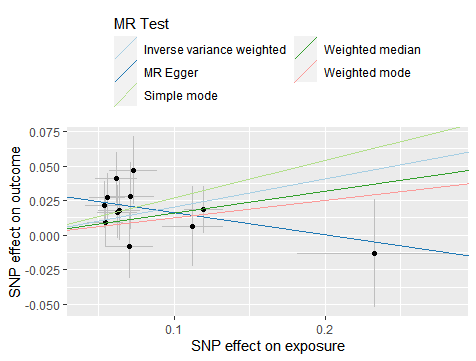** | **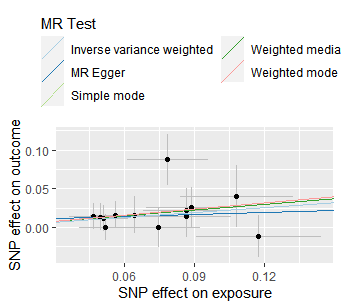** | **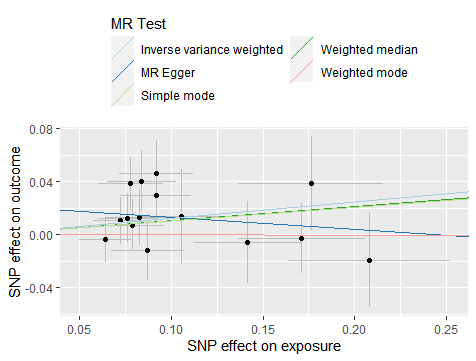** |
| **Pneumonia** | | |
| *Anaerotruncus* | *Barnesiella* | *Lachnospiraceae_NC2004_group* |
| **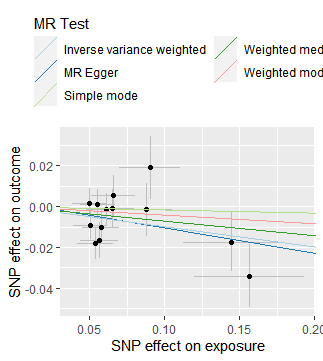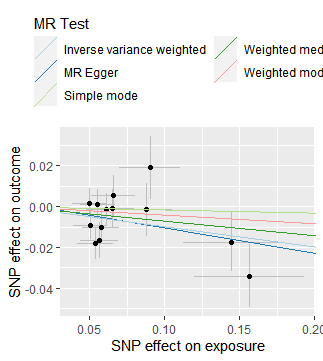** | **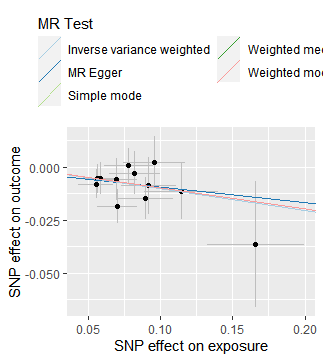** | **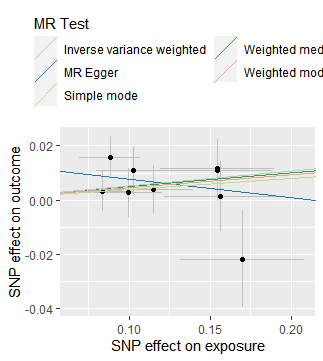** |

Supplementary Figure S2. Scatter plots for the causal association between gut microbiota and LRTIs in forward MR analysis of discovery dataset.

| **Bronchiectasis** | | |
| --- | --- | --- |
| *Bifidobacterium* | *Eubacterium_brachy_group* | *Eubacterium_ventriosum_group* |
| 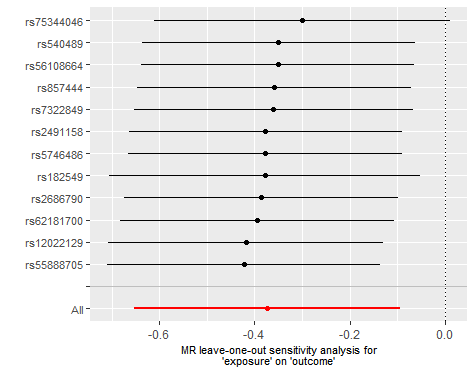 | 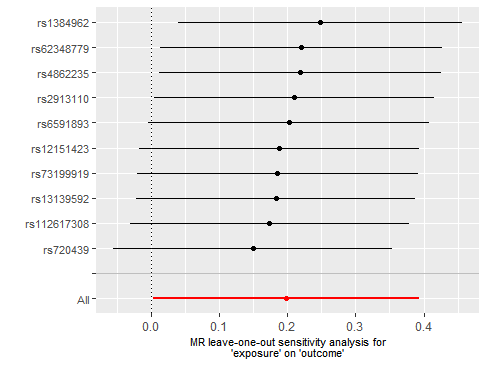 | 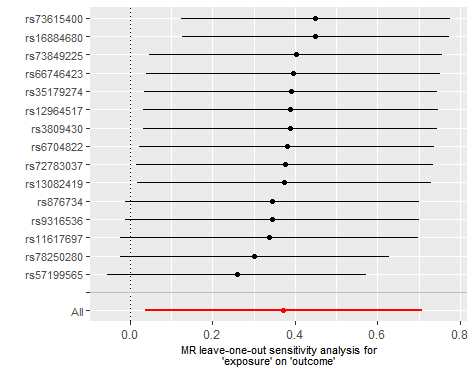 |
| *LachnospiraceaeFCS020_group* | *Parabacteroides* | *Peptococcus* |
| 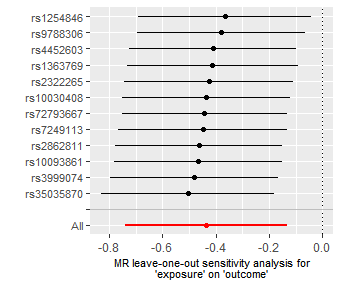 | 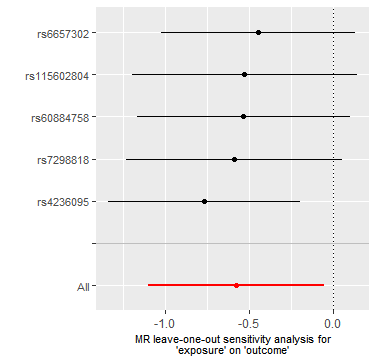 | 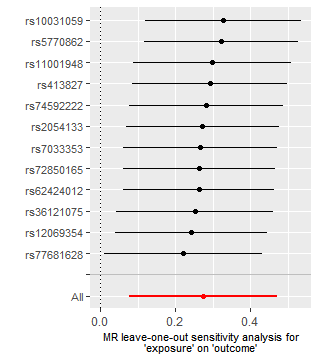 |
| **Acute bronchiolitis** | | |
| *Clostridium_sensu_stricto* | *Faecalibacterium* | *Haemophilus* |
| **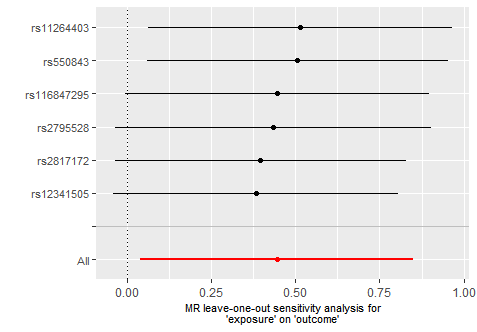** | **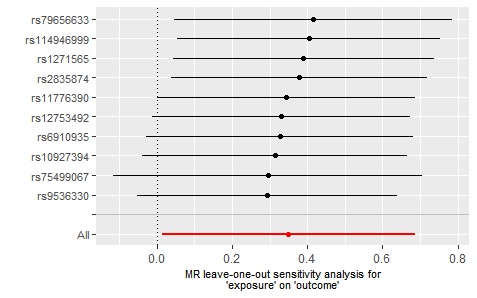** | **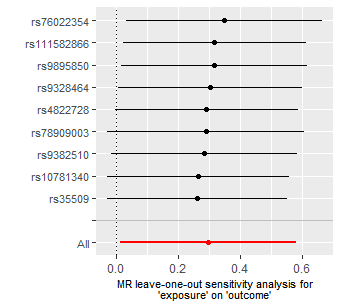** |
| *Oxalobacter* | *Tyzzerella* | *Veillonella* |
| 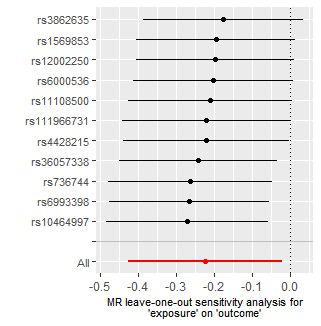 | 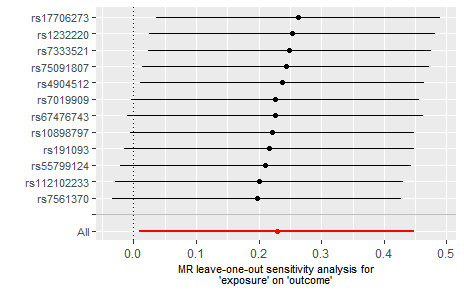 | 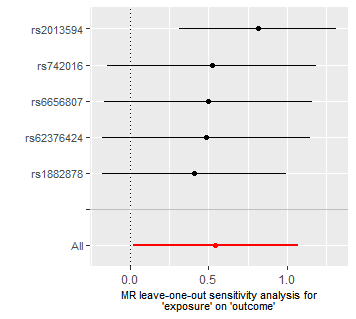 |
| **Acute bronchitis** | | |
| *Escherichia_Shigella* | *Eubacterium_fissicatena_group* | *Oxalobacter* |
| **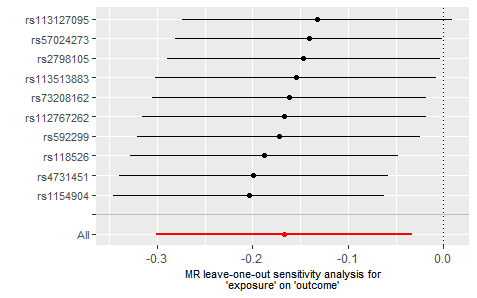** | **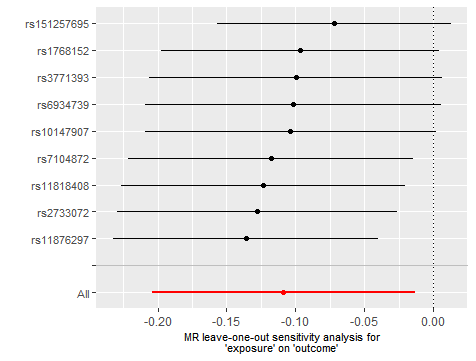** | **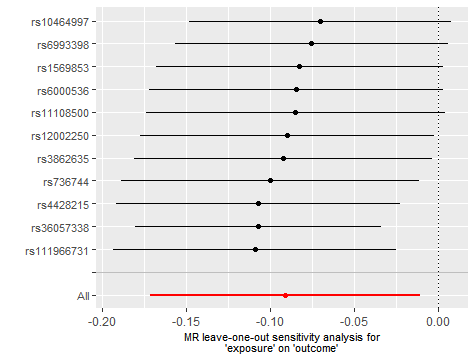** |
| **Influenza** | | |
| *Bifidobacterium* | *Lachnoclostridium* | *Prevotella_9* |
| **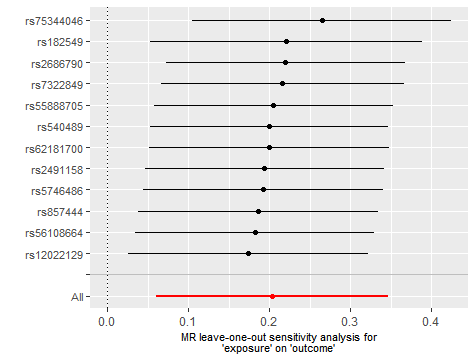** | **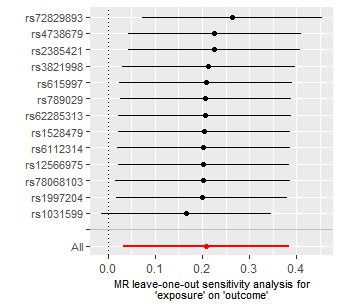** | **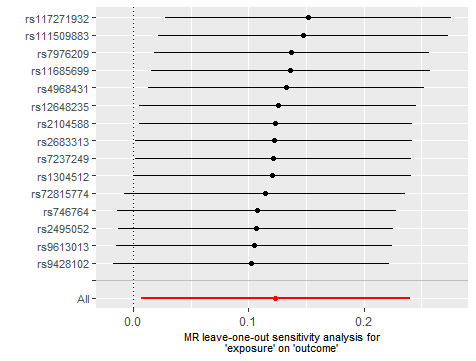** |
| **Pneumonia** | | |
| *Anaerotruncus* | *Barnesiella* | *Lachnospiraceae_NC2004_group* |
| **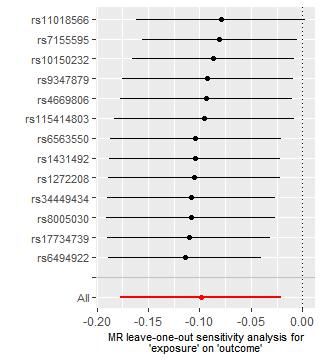** | **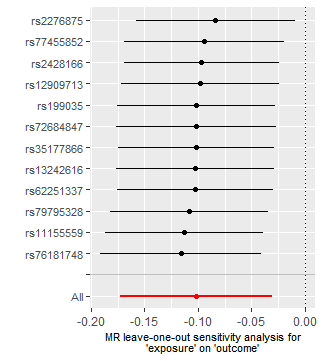** | **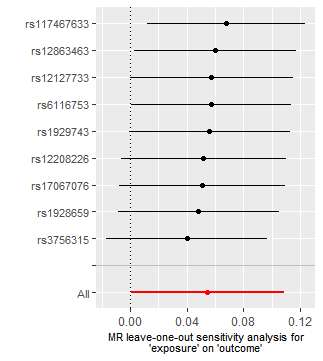** |

Supplementary Figure S3. Leave-one-out plots for the causal association between gut microbiota and LRTIs in forward MR analysis of discovery dataset.
